# Exosomal miR-370-3p increases the permeability of blood-brain barrier in ischemia/reperfusion stroke of brain by targeting MPK1

**DOI:** 10.18632/aging.204573

**Published:** 2023-03-08

**Authors:** Caifeng Gu, Weichun Mo, Kunlun Wang, Mingqiang Gao, Junfeng Chen, Feng Zhang, Jie Shen

**Affiliations:** 1 Center of Emergency and Intensive Care Unit, Jinshan Hospital, Fudan University, Shanghai, China

**Keywords:** stroke, blood-brain barrier, ischemia/reperfusion, exosomes, mir-370-3p

## Abstract

Ischemia/reperfusion (I/R) damage induced by stroke poses a serious hazard to human life, while mechanism of blood-brain barrier (BBB) dysfunction is still unknown. To imitate stroke induced ischemia conditions *in vivo*, the rat model of cerebral I/R damage was created by middle cerebral artery occlusion (MCAO). *In vitro*, the rat microvascular endothelial cell line bEND.3 was subjected to oxygen-glucose deprivation/reperfusion (OGD/R). Evans blue was used to evaluate the permeability of the blood-brain barrier (BBB). To evaluate gene expression at the mRNA and protein levels, researchers used real-time PCR and western blotting. Infarct volume and BBB permeability were considerably higher in cerebral (I/R) animals than in the Sham group. Exosomal miR-370-3p expression was shown to be higher in the brains of I/R injured rats and OGD/R treatment bEND.3. The BBB permeability was considerably increased when miR-370-3p was downregulated in OGD/R pretreated bEND.3. miR-370-3p regulates MAPK1 expression by targeting it. In bEND.3, OGD/R therapy increased BBB permeability substantially. OGD/R was inhibited by miR-370-3p mimic transfection, while miR-370-3p mimic was abolished by co-transfection with MAPK1 overexpression lentivirus. In cerebral I/R damage, exosomal miR-370-3p targets MAPK1 and aggregates BBB permeability.

## INTRODUCTION

According to current clinical statistics, the morbidity and mortality rates of stroke are still high [1]. The prevention and treatment strategies are still falling short of what is required [2]. Cerebral ischemia/reperfusion (I/R) damage caused by ischemic stroke is a severe condition that can be life-threatening. Pathophysiological mechanisms during the stroke are complex and variable. A prior study indicated that NADPH oxidase was aberrant in cerebral I/R damage [3]. It has been reported that cell excitotoxicity, inflammatory response, oxidative stress, and cell death were all involved in mediating brain injury [4]. Even though a lot of work has been put into exploring the mechanisms during cerebral I/R injury's process, the targeted treatment for brain damage is still not completely understood.

A highly selective permeability barrier, the blood-brain barrier (BBB), maintains the microenvironment of the brain by separating the blood from the brain's extracellular fluid [5]. The cerebral I/R damage can alter BBB disruption, increase cerebral vascular permeability and worsen cerebral infarction during stroke [6]. Therefore, maintaining the BBB's integrity is crucial for preserving brain function and inhibiting worse progression. Thus, identifying the process of BBB integrity breaches and understanding the specific mechanisms have been the subject of previous research [7]. These studies have identified numerous crucial regulatory mechanisms, including microRNAs (miRNAs) and several signaling pathways, which are essential for maintaining BBB integrity during stroke [8, 9].

The small non-coding single-stranded RNA, microRNAs (miRNAs), which have a length of 18 to 25 nucleotides, are crucial for controlling transcription by targeting downstream genes [10]. According to mounting evidence, miRNA dysregulation is closely associated with neurological illnesses, including stroke [11]. miRNAs regulate angiogenesis, apoptosis, and oxidative stress in both endothelial cells and supporting cells during ischemic stroke, according to previous studies [12]. A recent study showed that miR-370 is elevated during hepatic reperfusion and worsens hepatic injury via regulating the NF-B pathway [13]. Exosomal miR-370-3p also offered a possibility to be treated as a biomarker or a potential treatment for several diseases, including cerebrovascular disease [14, 15]. However, the mechanisms of miR-370-3p affect cerebral I/R during stroke remain unclear. In the current study, we sought to elucidate how miR-370-3p affects the BBB permeability in cerebral I/R stroke and to investigate the underlying mechanisms.

## MATERIALS AND METHODS

### Cells

DMEM supplemented with 10% FBS was used to cultivate the mouse microvascular endothelial cell line bEND.3, which was then kept at 37° C with a humidity of 95% for 24 hours. By moving cultures to glucose-free media pre-equilibrated with 95% N2 and 5% CO2 for 6 hours, bEND.3 was subjected to oxygen-glucose deprivation (OGD) therapy to simulate the ischemia state. The cells were then moved to a regular medium and recovered at 37° C in a humidified environment of 5% CO2 and 95% air in preparation for reperfusion.

### Animals

The Ethics Committee of Jinshan Hospital, Fudan University, approved this study. Male Sprague-Dawley (SD) rats weighing 250±30g were raised in ventilated cages with regular feedings and access to clean water. The Guidelines for the Care and Use of Laboratory Animals were followed in all experiments. According to a previous study, rats were anesthetized using 5% isoflurane, and the middle cerebral artery was blocked with a 3/0 monofilament nylon suture. Rats were fed in an intensive care incubator after surgery, with the temperature set at 37° C. Then, the suture was removed to induce reperfusion after two hours [16]. After the rats were deeply anesthetized, miR-370-3p mimics, miR-370-3p inhibitor, lentivirus, or pre-NC were injected into lateral ventricles. Three days later, the rats received MCAO surgery.

### Exosome isolation

According to previous studies, differential centrifugation was applied to separate exosomes from Cerebral I/R models. To remove cellular debris, cell culture media was centrifuged at 300 g for 10 min, then at 2000 g for 20 min, and finally at 10,000 g for 30 min at 4° C. To pellet exosomes, samples were ultracentrifuged twice for 90 min at 10,000 g, with PBS being used to resuspend between spins. Western blotting and transmission electron microscopy were used for examination.

### Evans blue staining

To evaluate the permeability of BBB, Evans blue dye was used for fluorescence detection. After 2 hours of ischemia, 2% Evans blue in PBS (4 ml/kg, i.v.) was administered for 24 hours. We used a fluorescent plate reader with OD = 620 nm (excitation) and 680 nm (emission) to measure dye concentrations.

### Measurement of infarct volume

Infarct brain tissues were isolated after the rats were killed, and infarct volume was measured according to a previous study [17]. The sliced brain was fixed in 4% paraformaldehyde at 37° C and stained with 1% 2,3,5-triphenyl tetrazolium chloride (TTC) for 30 min. Infarct volume was quantified using Image J Software.

### Real-time PCR

Total RNA was isolated using TRIzol reagent, and the RNA quality was determined using A260/A280 with a value between 1.8 and 2.0. According to the manufacturer’s instructions, Reverse Transcription Kit was used for obtaining cDNA, and then a TaqMan RNA assay was performed. The data were analyzed using the 2^−ΔΔCt^ method. The primers sequences were list as follows: miR-370-3p F: 5’-GAGACCAGGTCACGTCTCTG-3’; R: 5’-ACAGACAAACCAGGTTCCACC-3’. U6 F: 5’- GAGACCAGGTCACGTCTCTG-3’; R: 5’-ACAGACAAACCAGGTTCCACC-3’. MAPK1 F: 5’-AACACAACAAAAAGCCGCCC-3’; R: 5’-TGGTACTCAGTGGGGGTGAT-3’. β-actin F: 5’-CATGTACGTTGCTATCCAGGC-3’; R: 5’-CTCCTTAATGTCACGCAC GAT-3’.

### Western blot

The protein was extracted utilizing RIPA Lysis buffer, and the BCA method was used to calculate the protein concentration. The protein was deposited onto the PVDF membrane and treated with the primary antibodies (CD63, 1:1000; TGS101, 1:1000; α-Tubulin, 1:1000) after being separated into equal amounts using 10% SDS-PAGE at 4° C overnight. The membrane was then washed with PBS before incubating with the second antibody for 1 hour at room temperature. The bands were analyzed with the Bio-Image Analysis System and quantified with an enhanced chemiluminescence kit.

### Luciferase reporter assay

To create MAPK1-3′UTR-WT and MAPK1-3′UTR-Mut, the MAPK1 3′UTR fragment that contained the miR-370-3p binding site was amplified and cloned into the luciferase vector. The miR-370-3p mimic/inhibitor and the vector were co-transfected into cells using Lipofectamine 2000 following the manufacturer's instructions. Dual-Luciferase Reporter Assay was used to evaluate relative luciferase activity (Promega, USA).

### Statistical analyses

Data were presented as the mean ± standard deviation (SD) and analyzed using PASW Statistics 18.0 and GraphPad Prism 9.0. Comparisons were carried out using Student's t-tests or one-way analyses of variance (ANOVAs), followed by Tukey's or Dunnett's post hoc tests. The correlation between miR-370-3p expression levels and BBB permeability was evaluated using χ^2^-tests. The Pearson correlation coefficient examined the association between miR-370-3p expression and MAPK1. P<0.05 was considered the threshold of significance.

### Data availability

The raw data supporting the conclusions of this article will be made available by the authors, without undue reservation.

## RESULTS

### miR-370-3p increased in the brain tissues of cerebral I/R rats

**Figure 1:**
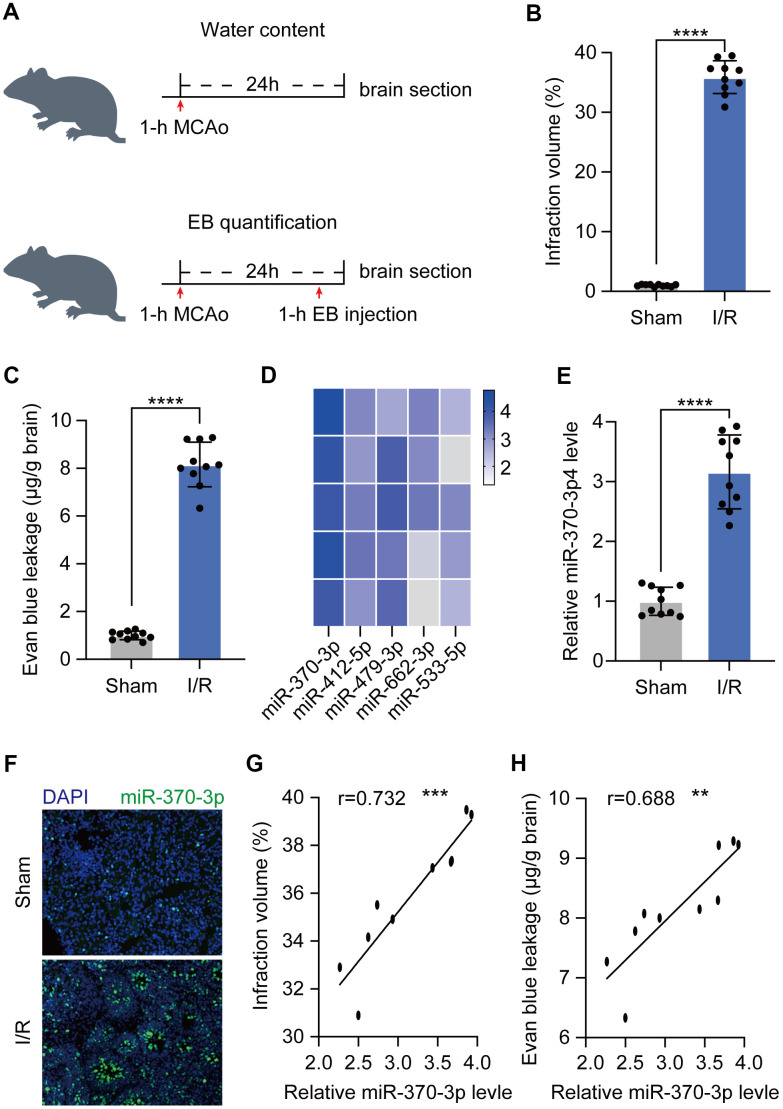
**Expression of miR-370-3p in the cerebral I/R rats.** (**A**) Experimental design to measure BBB leakage in cerebral I/R rats (n=10). (**B**) Infarct volume was assessed using 2,3,5-triphenyltetrazolium chloride (TTC) staining. (**C**) BBB permeability was evaluated by Evan’s blue assay. (**D**) The top 5 most highly expressed mRNAs in I/R rats based on RNA sequencing data. (**E**) The relative expressions of miR-370-3p in the cerebral I/R rats were determined by qPCR. (**F**) miR-370-3p levels were assessed by immunofluorescent staining. (**G**) Correlation between miR-370-3p and infraction volume. (**H**) Correlation between miR-370-3p and BBB permeability. ** p<0.01, *** p<0.005, **** p<0.001.

To evaluate the extravasation of blood components into brain parenchyma following BBB disruption, a rat cerebral I/R model was developed and validated in both MCAO-induced ischemic hemispheres and nonischemic hemispheres (sham) (n=10) (Figure 1A). In cerebral I/R rats (n=10), 2 h of ischemia followed by 24 h of reperfusion elicited a cerebral infarction that was statistically significant when compared to sham animals (n=10) (Figure 1B). Before sacrifice, Evan's blue was given intravenously, and water content was assessed 24 hours following 1 hour of acute focal ischemia. This test demonstrated that cerebral I/R rats had a much greater BBB permeability than the control group (Figure 1C). To investigate the underlying processes, RNA sequencing was used to assess the expression profile of miRNA and qRT-PCR to confirm the levels of the miR-370-3p in cerebral I/R rats and matched normal tissue samples (Figure 1D, 1E). According to immunofluorescence images, cerebral I/R tissues displayed higher levels of miR-370-3p than the control group (Figure 1F). These findings suggested that miR-370-3p significantly increased in cerebral I/R rats. Further correlation analysis revealed that miR-370-3p is positively associated with both the magnitude of cerebral infarction and the permeability of the BBB (Figure 1G, 1H). Our findings revealed that miR-370-3p increased in cerebral I/R rats, indicating that it plays a crucial role in controlling BBB permeability after stroke.

### Exosomes exhibit miR-370-3p up-regulation in cerebral I/R rats

**Figure 2:**
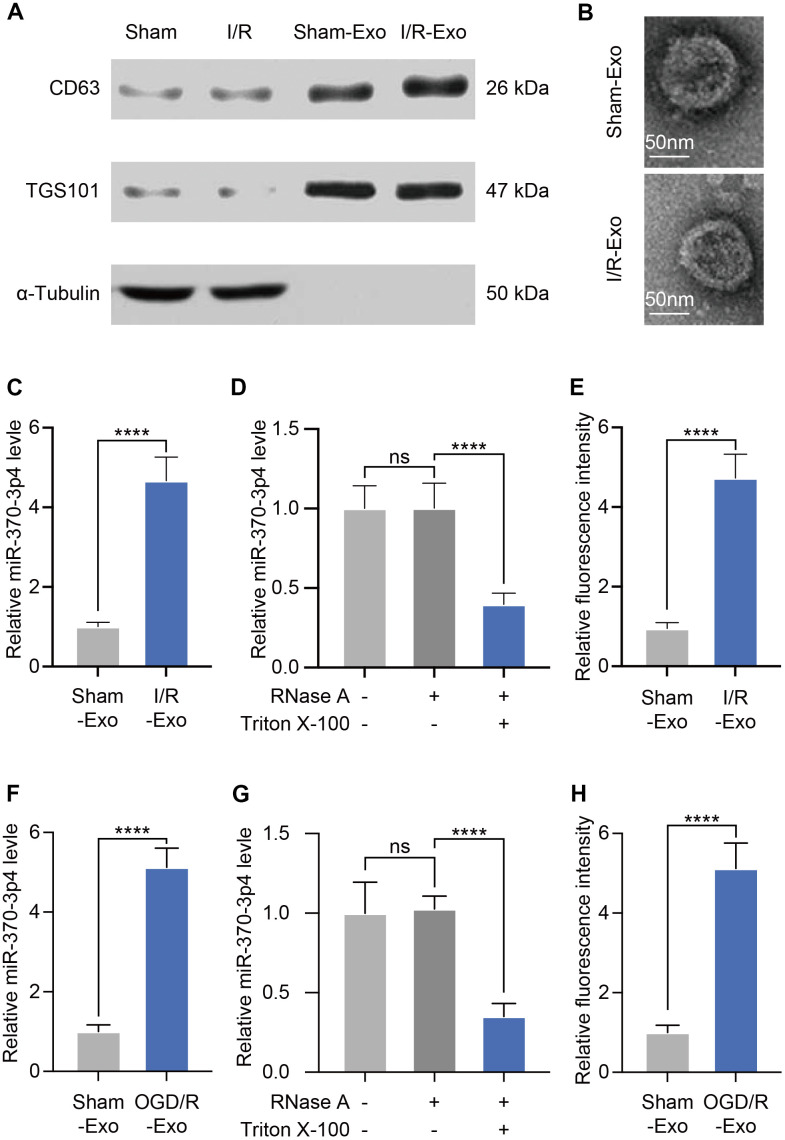
**Exosomes derived from cerebral I/R rats exhibit miR-370-3p upregulation.** (**A**) The levels of CD63, TSG101, and α-Tubulin were tested by western blotting. (**B**) Cerebral MVs-derived exosomes were analyzed via electron microscopy (scale bar, 50 nm). (**C**) Relative miR-370-3p expression in exosomes derived from MVs were tested by qPCR. (**D**) After treatment with 2 mg/ml RNase alone or combined with 0.1% Triton X-100, miR-370-3p level was analysed using qPCR in the culture medium of MVs. (**E**) miR-370-3p expression was assessed by fluorescence *in situ* hybridization (FISH) in cerebral I/R rats and healthy rats. (**F**) miR-370-3p expression levels in bEND.3, were assessed via qPCR. (**G**) After treatment with 2 mg/ml RNase alone or combined with .1% Triton X-100, miR-370-3p level was analysed using qPCR in the culture medium of bEND.3. (**H**) Quantitative analysis indicated that relative fluorescence intensity in bEND.3. ** p<0.01, *** p<0.005, **** p<0.001.

Exosomes are microscopic, membrane-enclosed particles secreted by all cell types in the brain to facilitate intercellular communication. (Exosomes and Their Role in the Life Cycle and Pathogenesis of RNA Viruses). We subsequently harvested exosomes from cerebral microvessels (MVs) via ultracentrifugation. Western blotting assays revealed that extracted substances exhibit high quantities of exosomal marker proteins (CD63 and TSG101) but no α-tubulin, which was in line with prior reports (Figure 2A). Subsequent TEM analysis verified that these particles were 80–120 nm in diameter and had a double-layered membrane (Figure 2B). These results validated the effective isolation of exosomes from cerebral I/R rats. Exosomes usually contain substantial amounts of microRNAs, enabling their transfer across cells. We tried to identify the expression of miR-370-3p in brain cells-derived exosomes and discovered that it is highly up-regulated in cerebral I/R rats (Figure 2C). To investigate the existence of extracellular miR-370-3p, we treated MVs with Triton X-100 and RNase. It was found that processing with RNase did not affect the level of miR-370-3p in the culture medium. However, the combination of Triton X-100 and RNase significantly reduced the level of miR-370-3p, indicating that extracellular miR-370-3p was predominantly membrane-bound as opposed to directly released (Figure 2D). We further assessed miR-370-3p in I/R samples using FISH and found that it was up-regulated in I/R samples compared to healthy rats (Figure 2E). Evaluation of miR-370-3p levels in bEND.3-derived exosomes revealed that it was considerably up-regulated after OGD/R exposure (Figure 2F). Triton X-100 and RNase also significantly eliminated the level of miR-370-3p in the culture medium (Figure 2G). Quantitative analysis indicated that relative fluorescence intensity was increased in OGD/R bEND.3 cells (Figure 2H). Together, these data suggest that exosomes exhibit miR-370-3p up-regulation in cerebral I/R rats.

**Figure 3:**
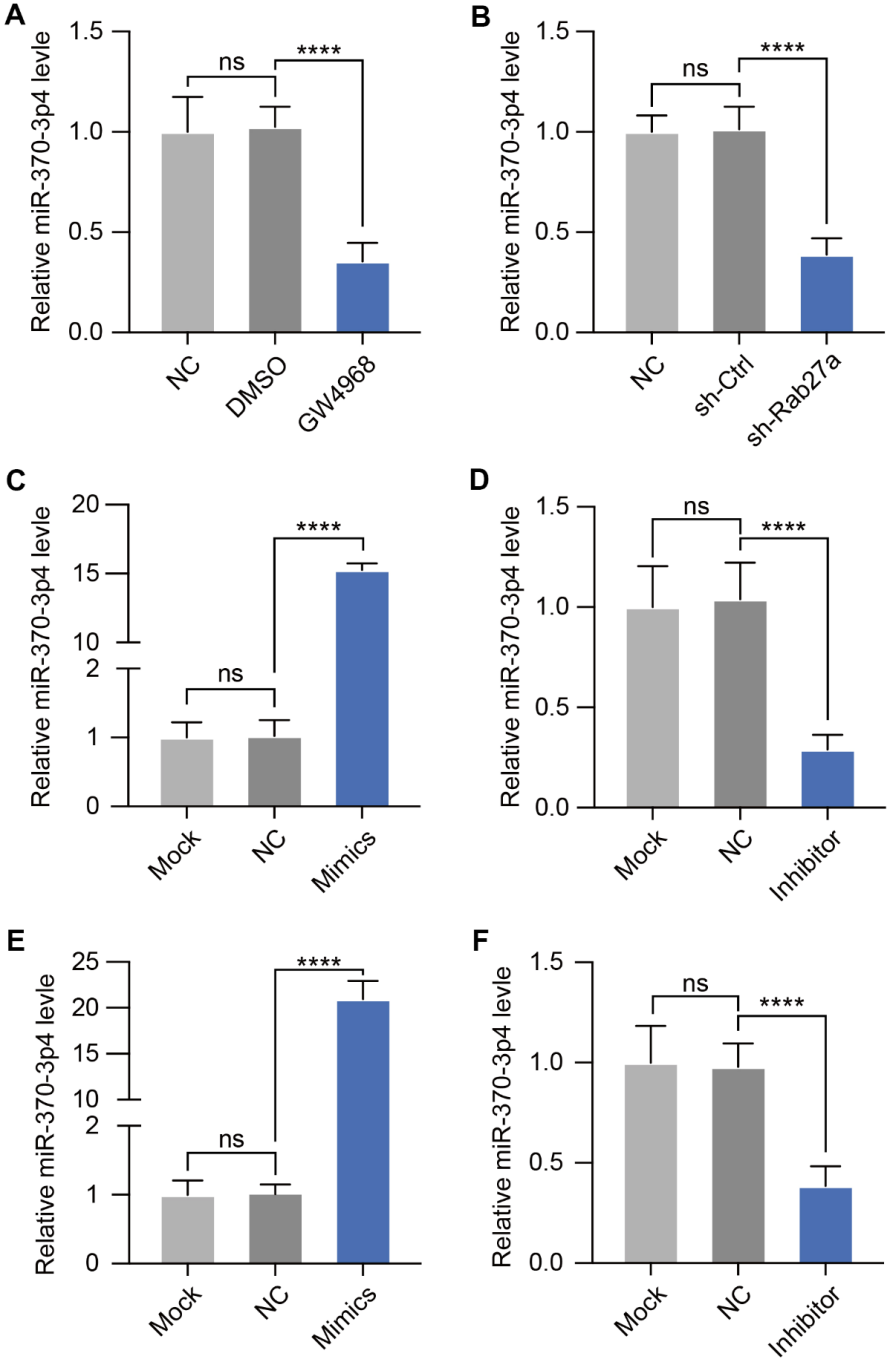
**GW4968 and Rab27a silencing down-regulate miR-370-3p in cerebral MVs.** (**A**) Following co-culture with exosomes treated with GW4869, miR-370-3p was analyzed in bEND.3 cells. (**B**) After Rab27a silencing, miR-370-3p levels in bEND.3 cells were tested by qPCR. (**C**, **D**). miR-370-3p levels in bEND.3 cells were measured via qPCR following miR-370-3p mimic or inhibitor treatment. (**E**, **F**) Cerebral MVs from I/R rats were collected and then transfected with miR-370-3p mimic or inhibitor. miR-370-3p expression in exosomes from these cells then being assessed via qPCR. ns, not significant, ** p<0.01, *** p<0.005, **** p<0.001.

To investigate whether miRNA is transferring in cerebral MVs via exosome transportation, we further treated bEND.3 cells with an exosome inhibitor (GW4968) and then measured the expression of miR-370-3p. qPCR assays revealed that GW4968 significantly decreased miR-370-3p levels in bEND.3 cells (Figure 3A). It has been found that alteration of the Rab27a gene regulates the effectiveness of exosome secretion. Thus, we treated bEND.3 cells with shRab27a for 24 hours showed a substantial decrease in miR-370-3p (Figure 3B). Then, we assessed levels of miR-370-3p inside bEND.3 after miR-370-3p mimic or inhibitor treatment and discovered that miR-370-3p mimic therapy significantly increased the expression of this miRNA within these cells, and inhibitor down-regulated miR-370-3p (Figure 3C, 3D). The levels of miR-370-3p within exosomes derived from MVs of cerebral I/R rats were also assessed by qPCR following miR-370-3p mimic or inhibitor treatment (Figure 3E, 3F). These results indicated that cerebral MVs derived miR-370-3p are primarily delivered through exosome-mediated mechanisms.

### miR-370-3p was able to aggravate I/R-induced BBB disruption

**Figure 4:**
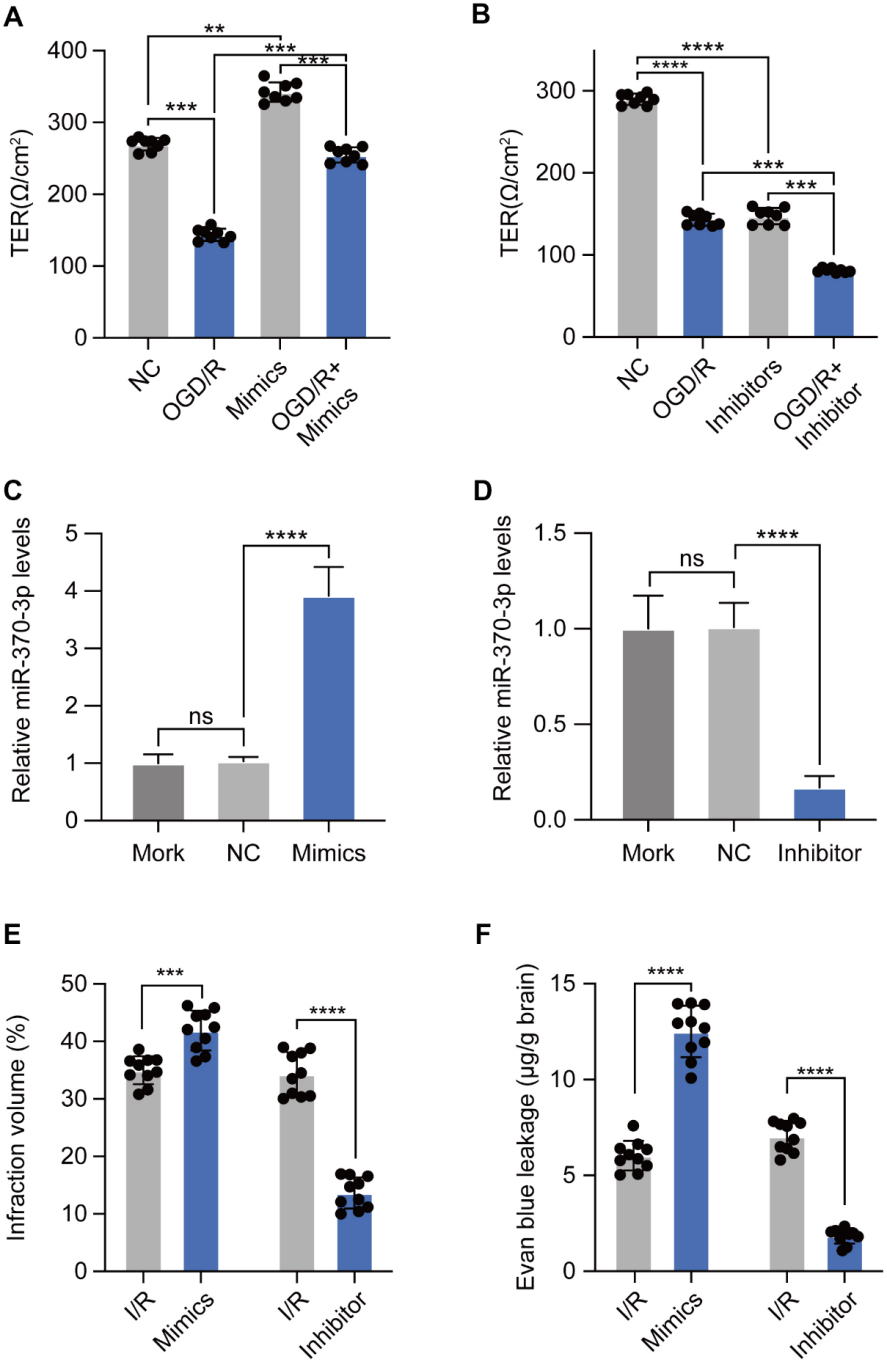
**miR-370-3p aggravates I/R-induced BBB disruption.** (**A**, **B**) The transendothelial electrical resistance of bEND.3 cells was measured using Millicell-ERS electrical resistance system. (**C**, **D**) The relative expressions of miR-370-3p in cerebral I/R rats with miR-370-3p mimics or inhibitors treatment. (**E**, **F**) Infraction volume and Evan blue leakage were used to evaluate BBB permeability with miR-370-3p mimics or inhibitor treatment. ns, not significant, ** p<0.01, *** p<0.005, **** p<0.001.

To explore the roles of miR-370-3p in BBB disruption of brain endothelial cells, transendothelial electrical resistance was used to evaluate BBB integrity in bEND.3 cells. Inhibition of miR-370-3p effectively reduced the transendothelial electrical resistance value of bEND.3 cells during OGD/R (Figure 4A). Consistently, overexpression of miR-370-3p could enhance the transendothelial electrical resistance value during OGD/R (Figure 4B). These findings suggested that OGD/R, at least in part, caused BBB disruption by suppressing miR-370-3p. *In vivo*, pre-NC, miR-370-3p mimics or inhibitors were utilized to regulate the levels of miR-370-3p in MVs As expected, transfection of miR-370-3p mimics elevated the expression level of miR-370-3p and inhibitors significantly inhibited the expression level of miR-370-3p (Figure 4C, 4D). Overexpression of miR-370-3p increased the BBB infarct volume of cerebral I/R rats (n=10), and inhibiting miR-370-3p reduced the infarct volume (n=10) (Figure 4E). Additionally, BBB permeability of cerebral I/R rats markedly increased after receiving miR-370-3p mimics. In contrast, BBB permeability decreased in cerebral I/R rats after receiving miR-370-3p inhibitors (Figure 4F). Together, these results showed that miR-370-3p overexpression exacerbates I/R-induced BBB disruption while miR-370-3p inhibition reduces it.

### miR-370-3p aggravates I/R-induced BBB disruption by suppressing MAPK1 expression

**Figure 5:**
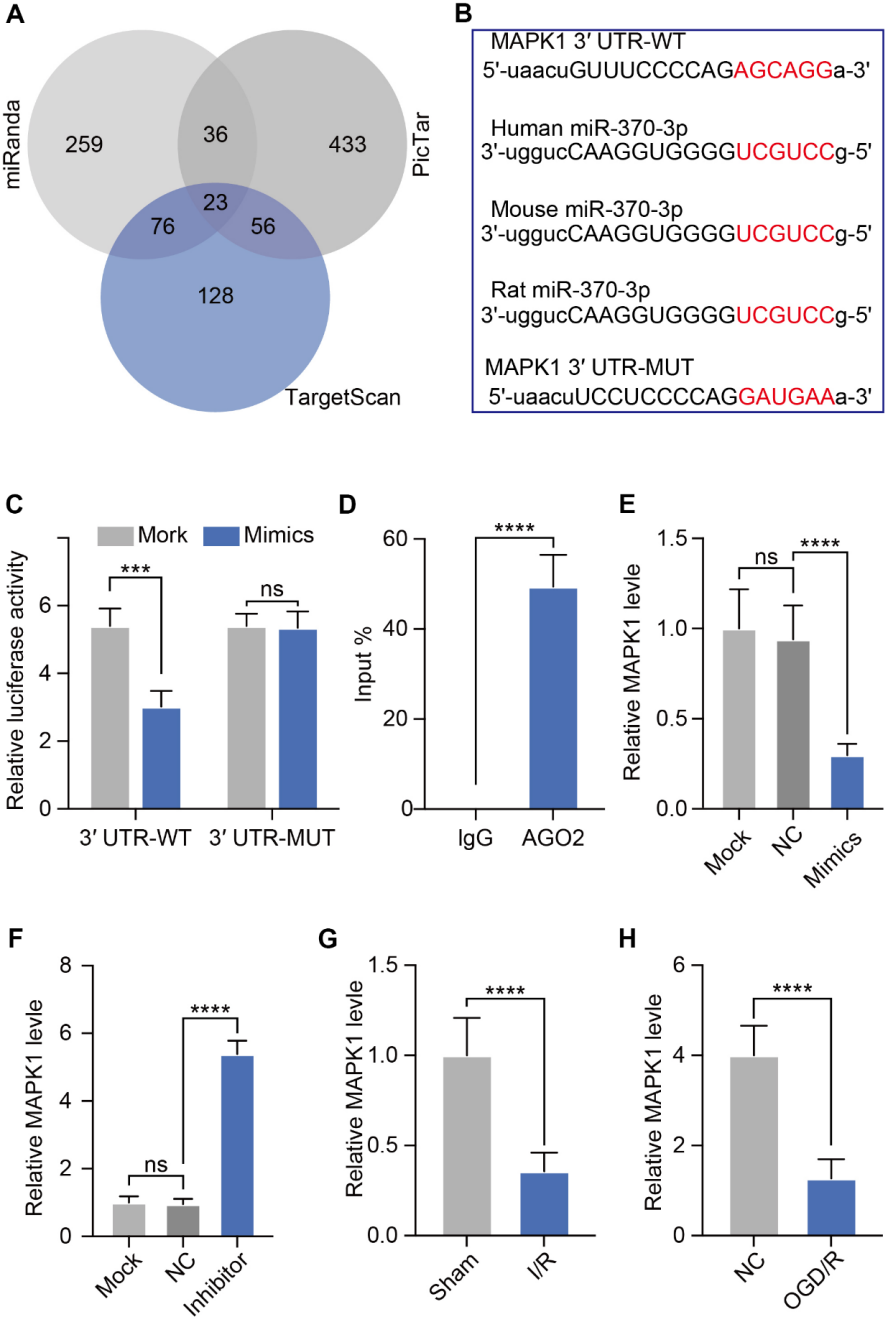
**miR-370-3p directly targets MAPK1.** (**A**) Candidate genes from three databases were shown in a Venn diagram. (**B**) Luciferase reporter constructs with WT or mutant (MUT) MAPK1 3'-UTR binding sites are shown schematically. (**C**) Following co-transfection of miR-370-3p mimics with WT or MUT reporter plasmids, luciferase activity in bEND.3 cells was assessed. (**D**) Interactions between MAPK1 and miR-370-3p were tested using RNA immunoprecipitation. (**E**, **F**) Relative MAPK1 expression in bEND.3 cells with miR-370-3p mimics or miR-370-3p inhibitor treatment. (**G**, **H**) Levels of MAPK1 in both cerebral I/R rats and OGD/R in bEND.3 cells.

We next used bioinformatics analysis to identify possible targets of miR-370-3p in order to understand the processes by which this miRNA may affect BBB of I/R development. The potential miR-370-3p target genes were predicted using the TargetScan, PicTar, and MiRanda databases. There were 23 target genes in the intersection of these three databases, as seen in the Venn diagram (Figure 5A). MAPK1 was chosen as a possible target of miR-370-3p by considering differential expression, pathways, and literature. We also found that various MAPK1 sequences of different species were homologous. A candidate miR-370-3p binding site was identified within the MAPK1 3′-UTR (Figure 5B). We next designed luciferase vectors containing WT or mutant (MUT) copies of the MAPK1 3′-UTR region to validate the capacity of miR-370-3p in the regulation of MAPK1 expression. Co-transfection of miR-370-3p mimics and MAPK1 plasmids reduced luciferase activity in the WT group but not in the MUT group (Figure 5C). Additionally, co-precipitation of miR-370-3p with MAPK1 was validated by RIP studies, indicating a strong interaction between these two targets (Figure 5D). These results, therefore, supported that miR-370-3p directly suppresses MAPK1 expression in a sequence-specific manner. To further confirm the regulatory relationship between miR-370-3p and MAPK1 in bEND.3 cells, we treated bEND.3 cells with miR-370-3p inhibitor or miR-370-3p mimics. The results showed that miR-370-3p inhibitor significantly increased MAPK1 and miR-370-3p mimics decreased MAPK1 in bEND.3 cells (Figure 5E, 5F). These results thus validated MAPK1 as a miR-370-3p target gene. Furthermore, we assessed the level of MAPK1 using qPCR and discovered that it was elevated in both cerebral I/R rats and OGD/R in bEND.3 cells (Figure 5G, 5H).

**Figure 6:**
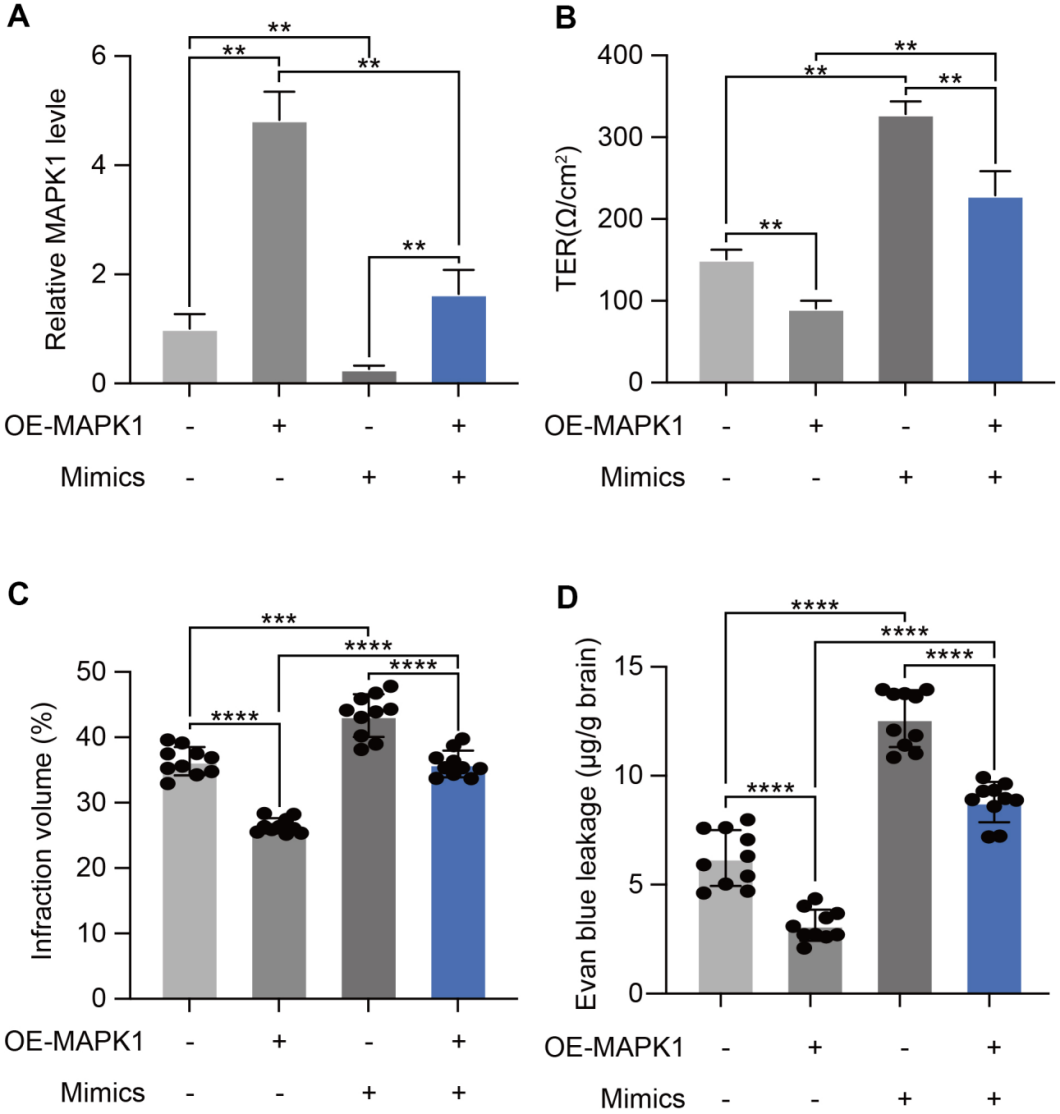
**miR-370-3p aggravated I/R-Induced BBB disruption by targeting MAPK1.** (**A**) qPCR demonstrating that miR-370-3p regulates MAPK1 expression. (**B**) Transendothelial electrical resistance assessments was implemented to analyze BBB disruption. (**C**, **D**) Cellular proliferation following miR-370-3p mimic transfection and/or MAPK1 overexpression was assessed via infarct volume and BBB permeability in brain tissues of cerebral I/R rats. ** p<0.01, *** p<0.005, **** p<0.001.

We next carried out rescue evaluations to determine the degree to which miR-370-3p-mediated MAPK1 down-regulation increases I/R-Induced BBB breakdown. MAPK1 levels were tested by qPCR in bEND.3 cells after miR-370-3p mimics and OE-MAPK co-transfection. The results showed that MAPK1 was up-regulated relative to the levels observed in cells with miR-370-3p mimics co-transfection and negative control (Figure 6A). Transendothelial electrical resistance measurements showed that MAPK1 overexpression prevented BBB breakdown, which was caused by miR-370-3p (Figure 6B). By co-transfecting with miR-370-3p mimics and either OE-MAPK1 or NC lentivirus, we were able to assess the effects of miR-370-3p and MAPK1 *in vivo*. In the MVs of cerebral I/R rats (n=10), MAPK1 overexpression decreased infarct volume and BBB permeability, whereas miR-370-3p mimics co-transfection reversed these effects (Figure 6C, 6D). These results supported the hypothesis that MAPK1 is a direct miR-370-3p target gene that causes a worsening BBB disruption during cerebral I/R.

## DISCUSSION

In several studies, BBB permeability in cerebral I/R damage has been widely examined [18]. The primary structural component of BBB is made up of endothelial cells, which are damaged during cerebral I/R. It has been reported that the cerebral I/R significantly increased the permeability of BBB by destroying the structural constituent of endothelial cells [19]. However, the mechanisms of cerebral I/R results in increased permeability of BBB are still unclear. As such, there is an obvious need to more fully discover the strategies to guide better patient diagnosis and treatment. In our present study, the results revealed that exosomal miR-370-3p aggravates the I/R-induced permeability of BBB by targeting MAPK1. Except that, miR-370-3p also specifically elevated TNF-a, which is closely related with brain apoptosis. At the BBB, claudin-5 is the most dominant claudin in endothelial cells. Thus, whether miR-370-3p aggravates the permeability of BBB need further exploration.

According to recent research, the pathophysiological alterations that occur during cerebral I/R are mostly determined by exosomal miRNAs. MiR-424 prevents brain damage in hypoxic conditions by reducing microglia activation [20]. Cardiac I/R damage was caused by MiR-21 by controlling the PTEN/Akt signaling pathway [21] and it was an essential biomarker for acute cerebral ischemia [18]. In addition, miR-124, miR-21, miR-23a and miR-181 were all involved in the cerebral ischemia [22–25]. Some of these miRNAs changed within 3-hours, which would be more clinically significant. It is interesting that plasma miR-370-3p also highly sensitive for early detection (6h) to BBB permeability. Even though numerous miRNAs have been examined to determine their roles in cerebral ischemia processes, specific mechanisms still need to be deeply explored. Therefore, further investigations into regulatory mechanisms of miRNAs are urgently necessary for the future. In the current study, we discovered that cerebral I/R injury rats had drastically altered miR-370-3p expression, suggesting that miR-370-3p may play a crucial role in cerebral I/R injury. Using the luciferase reporter assay, it was shown that the miR-370-3p binds MPK1 to control its expression. The expression of MPK1 was also significantly altered in the brain tissues of rats with cerebral I/R damage. Thus, we concluded that MPK1 is a target of miR-370-3p, which controls the I/R injury.

Moreover, the effects of MPK1 have been reported on various human diseases, including cerebral I/R. Additionally, MPK1 is responsible for interacting with JNK and p38 to regulate environmental stresses, such as oxidative stress and inflammatory cytokine, while ERK is usually involved in cell proliferation and growth. MPK1 can also stimulate transcription factors that regulate multiple genes involved in the survival or death of cells. Thus, the transcription factors play significant roles in pharmacological intervention and drug development. Collectively, those data indicated that exosomal miR-370-3p aggravated I/R-induced BBB disruption by targeting MPK1. It also emphasized the protective role of MPK1 on cerebral I/R injury.

## References

[1] Roger VL, Go AS, Lloyd-Jones DM, Adams RJ, Berry JD, Brown TM, Carnethon MR, Dai S, de Simone G, Ford ES, Fox CS, Fullerton HJ, Gillespie C, et al, and American Heart Association Statistics Committee and Stroke Statistics Subcommittee. Heart disease and stroke statistics--2011 update: a report from the American Heart Association. Circulation. 2011; 123:e18–209. 10.1161/CIR.0b013e3182009701 21160056 PMC4418670

[2] Kishimoto M, Suenaga J, Takase H, Araki K, Yao T, Fujimura T, Murayama K, Okumura K, Ueno R, Shimizu N, Kawahara N, Yamamoto T, Seko Y. Oxidative stress-responsive apoptosis inducing protein (ORAIP) plays a critical role in cerebral ischemia/reperfusion injury. Sci Rep. 2019; 9:13512. 10.1038/s41598-019-50073-8 31534168 PMC6751213

[3] Guo H, Jiang Y, Gu Z, Ren L, Zhu C, Yu S, Wei R. ZFP36 protects against oxygen-glucose deprivation/reoxygenation-induced mitochondrial fragmentation and neuronal apoptosis through inhibiting NOX4-DRP1 pathway. Brain Res Bull. 2022; 179:57–67. 10.1016/j.brainresbull.2021.12.003 34896479

[4] Ma MW, Wang J, Zhang Q, Wang R, Dhandapani KM, Vadlamudi RK, Brann DW. NADPH oxidase in brain injury and neurodegenerative disorders. Mol Neurodegener. 2017; 12:7. 10.1186/s13024-017-0150-7 28095923 PMC5240251

[5] Kadry H, Noorani B, Cucullo L. A blood-brain barrier overview on structure, function, impairment, and biomarkers of integrity. Fluids Barriers CNS. 2020; 17:69. 10.1186/s12987-020-00230-3 33208141 PMC7672931

[6] Zhang ZQ, Song JY, Jia YQ, Zhang YK. Buyanghuanwu decoction promotes angiogenesis after cerebral ischemia/reperfusion injury: mechanisms of brain tissue repair. Neural Regen Res. 2016; 11:435–40. 10.4103/1673-5374.179055 27127482 PMC4829008

[7] Ronaldson PT, Davis TP. Regulation of blood-brain barrier integrity by microglia in health and disease: A therapeutic opportunity. J Cereb Blood Flow Metab. 2020; 40:S6–24. 10.1177/0271678X20951995 32928017 PMC7687032

[8] Nian K, Harding IC, Herman IM, Ebong EE. Blood-Brain Barrier Damage in Ischemic Stroke and Its Regulation by Endothelial Mechanotransduction. Front Physiol. 2020; 11:605398. 10.3389/fphys.2020.605398 33424628 PMC7793645

[9] Lopez-Ramirez MA, Wu D, Pryce G, Simpson JE, Reijerkerk A, King-Robson J, Kay O, de Vries HE, Hirst MC, Sharrack B, Baker D, Male DK, Michael GJ, Romero IA. MicroRNA-155 negatively affects blood-brain barrier function during neuroinflammation. FASEB J. 2014; 28:2551–65. 10.1096/fj.13-248880 24604078

[10] Kim VN. MicroRNA biogenesis: coordinated cropping and dicing. Nat Rev Mol Cell Biol. 2005; 6:376–85. 10.1038/nrm1644 15852042

[11] Bhalala OG, Srikanth M, Kessler JA. The emerging roles of microRNAs in CNS injuries. Nat Rev Neurol. 2013; 9:328–39. 10.1038/nrneurol.2013.67 23588363 PMC3755895

[12] Yin KJ, Hamblin M, Chen YE. Angiogenesis-regulating microRNAs and Ischemic Stroke. Curr Vasc Pharmacol. 2015; 13:352–65. 10.2174/15701611113119990016 26156265 PMC4079753

[13] Zhu J, Zhu F, Song W, Zhang B, Zhang X, Jin X, Li H. Altered miR-370 expression in hepatic ischemia-reperfusion injury correlates with the level of nuclear kappa B (NF-κB) related factors. Gene. 2017; 607:23–30. 10.1016/j.gene.2016.12.026 28043920

[14] Mao J, Wang L, Wu J, Wang Y, Wen H, Zhu X, Wang B, Yang H. miR-370-3p as a Novel Biomarker Promotes Breast Cancer Progression by Targeting FBLN5. Stem Cells Int. 2021; 2021:4649890. 10.1155/2021/4649890 34475958 PMC8407987

[15] Wei Y, Zhang Q, An L, Fang G, Hong D, Jiao T, Yang H, Wang Z. Serum exosomal microRNA-370-3p and microRNA-196a-5p are potential biomarkers for the diagnosis and prognosis of hepatocellular carcinoma. Folia Histochem Cytobiol. 2022; 60:215–25. 10.5603/FHC.a2022.0019 35762276

[16] Fu K, Chen M, Zheng H, Li C, Yang F, Niu Q. Pelargonidin ameliorates MCAO-induced cerebral ischemia/reperfusion injury in rats by the action on the Nrf2/HO-1 pathway. Transl Neurosci. 2021; 12:20–31. 10.1515/tnsci-2021-0006 33552591 PMC7821419

[17] Mao C, Hu C, Zhou Y, Zou R, Li S, Cui Y, Tian W. Electroacupuncture Pretreatment against Cerebral Ischemia/Reperfusion Injury through Mitophagy. Evid Based Complement Alternat Med. 2020; 2020:7486041. 10.1155/2020/7486041 32963572 PMC7499311

[18] Fan F, Yang J, Xu Y, Guan S. MiR-539 Targets MMP-9 to Regulate the Permeability of Blood-Brain Barrier in Ischemia/Reperfusion Injury of Brain. Neurochem Res. 2018; 43:2260–7. 10.1007/s11064-018-2646-0 30276507

[19] Yang X, Liang J, Jia M, Yang T, Deng X, Wang P, Ren L, Gao S, Zuo Z, Pei D, Bi J, Wang P. β-1, 3-galactosyltransferase 2 ameliorates focal ischemic cerebral injury by maintaining blood-brain barrier integrity. Neurochem Int. 2021; 144:104976. 10.1016/j.neuint.2021.104976 33524473

[20] Liu P, Zhao H, Wang R, Wang P, Tao Z, Gao L, Yan F, Liu X, Yu S, Ji X, Luo Y. MicroRNA-424 protects against focal cerebral ischemia and reperfusion injury in mice by suppressing oxidative stress. Stroke. 2015; 46:513–9. 10.1161/STROKEAHA.114.007482 25523055

[21] Huang J, Qi Z. MiR-21 mediates the protection of kaempferol against hypoxia/reoxygenation-induced cardiomyocyte injury via promoting Notch1/PTEN/AKT signaling pathway. PLoS One. 2020; 15:e0241007. 10.1371/journal.pone.0241007 33151961 PMC7644004

[22] Liu X, Feng Z, Du L, Huang Y, Ge J, Deng Y, Mei Z. The Potential Role of MicroRNA-124 in Cerebral Ischemia Injury. Int J Mol Sci. 2019; 21:120. 10.3390/ijms21010120 31878035 PMC6981583

[23] Xu X, Kriegel AJ, Jiao X, Liu H, Bai X, Olson J, Liang M, Ding X. miR-21 in ischemia/reperfusion injury: a double-edged sword? Physiol Genomics. 2014; 46:789–97. 10.1152/physiolgenomics.00020.2014 25159851 PMC4280148

[24] Siegel C, Li J, Liu F, Benashski SE, McCullough LD. miR-23a regulation of X-linked inhibitor of apoptosis (XIAP) contributes to sex differences in the response to cerebral ischemia. Proc Natl Acad Sci USA. 2011; 108:11662–7. 10.1073/pnas.1102635108 21709246 PMC3136267

[25] Xu LJ, Ouyang YB, Xiong X, Stary CM, Giffard RG. Post-stroke treatment with miR-181 antagomir reduces injury and improves long-term behavioral recovery in mice after focal cerebral ischemia. Exp Neurol. 2015; 264:1–7. 10.1016/j.expneurol.2014.11.007 25433215 PMC4324354

